# Animal Models for Radiotherapy Research: All (Animal) Models Are Wrong but Some Are Useful

**DOI:** 10.3390/cancers13061319

**Published:** 2021-03-16

**Authors:** Karl T. Butterworth, Jacqueline P. Williams

**Affiliations:** 1 Patrick G. Johnston Centre for Cancer Research, Queen’s University Belfast, Belfast BT9 7AE, UK; 2 University of Rochester Medical Centre, University of Rochester, Rochester, NY 14642, USA; Jackie_Williams@urmc.rochester.edu

The distinguished statistician, George E.P. Box, stated that “All models are wrong but some are useful” [1]. Whilst this aphorism was originally intended to describe the utility of statistical models, it can be considered an equally accurate narrative for the use of animal models in biomedical research. Indeed, animal models have played a key role in advancing our understanding of tumour biology and response to cancer treatments, especially radiotherapy. Since the first report of the tissue-sparing effects from Regaud and Nogier in 1911 [2], where they assessed the outcome of dose fractionation using a ram testicular model, animal models have been considered invaluable experimental tools, furthering our understanding of radiotherapy response at the cell, tissue, and whole-organism levels.

The use of animal models in research has long been predicated on purported genetic, anatomical and physiological similarities of different model species to humans. Overall, the house mouse (*Mus musculus*) is the most frequently used animal model for experimental studies; in part, this is due to its genomic synteny with man, as well as sharing common organs, systems physiology, and many pathologies, although economics and relative ease of maintenance and breeding likely also have a significant influence on this choice. However, increasing concerns have been voiced, both from within the scientific community as well as from the public at large, regarding not only the ethical use of animals per se, but also the relevance and translation of findings to humans [3,4]. Indeed, from the radiobiology field, inbred mice have a close to two-fold difference in radiosensitivity compared to humans based on respective LD50/30 and LD50/60 after total body irradiation [5]. However, it appears that radiobiologic differences may be smaller when appropriate models are chosen and tested using clinically relevant exposures and regimens. In this series of original articles and review papers, we highlight some of the innovative experimental approaches that are being implemented in the laboratory, with the goal of offering a critical discussion on the translational relevance of animal models to human radiation response.

Brown and colleagues [6] provide an example of how the classical bench-to-bedside research paradigm can be reversed to bring clinical approaches back into the laboratory. Their study explores the use of a clinical fiducial marker in small animal image guided-radiotherapy (IGRT), demonstrating BioXmark as a tool to improve target delineation and reduce set-up errors with minimal impacts on the radiobiological response. Another powerful example of reverse translation is provided by Bolookat [7] who reports an integrated workflow for MRI-guided volumetric arc therapy (VMAT) in a rabbit tumour model of HNSCC. This study demonstrates the feasibility of developing a clinically-relevant workflow in a laboratory, with potential applications to examine long-term survival and evaluate disease recurrence with MR-guided VMAT.

Despite the major clinical advances that have been made in the delivery of conformal radiotherapy and image guidance, the risk of normal tissue complication continues to limit dose escalation and significantly impact quality of life following treatment. Innovative preclinical models are being developed to assess responses across a range of normal tissue. For example, Schlaak et al. [8] summarise current state-of-the-art preclinical models of radiation-induced cardiac toxicity, describing the progress in experimental irradiation techniques from whole thorax exposures to those using small animal IGRT. Using an interleukin-2 receptor gamma chain knockout (IL2RG^−/−^) rat model of T- and B-cell deficiency with image-guided whole-heart exposures, the group investigated the role of acquired immunity radiation-induced heart dysfunction [9]. Their study showed that IL2RG deficiency protects from cardiac hypertrophy, but contributes to cardiac dysfunction, highlighting the complex roles for adaptive immune cells in cardiac injury that vary depending on clinically relevant factors including dose, fractionation, sex, and genetic background. Importantly, the authors discuss the major differences in cardiac physiology between humans and different animal models that should be considered when interpreting data from preclinical studies.

Other examples in this edition include a paper from Kabarriti and colleagues [10], which describes a novel small animal IGRT model of hepatic irradiation. This approach was combined with single-photon emission computed tomography (SPECT) imaging for the functional assessment of early region-specific radiation-induced liver damage. The review from Farris et al. [11] covers current preclinical studies aimed at investigating the biological basis of radiation-induced musculoskeletal toxicity, another major source of morbidity in patients receiving radiotherapy. Furthermore, Pazzaglia and colleagues [12] provide a comprehensive review of the mechanisms and therapeutic implications of neurocognitive decline following radiotherapy, with an emphasis on radiation-induced stem cell injury and its functional consequences.

Craniospinal irradiation (CSI) involves the irradiation of the entire neuraxis, including the brain, spinal cord and extensions along the nerve roots, and is frequently used in the management of pediatric medulloblastoma. Stripay and colleagues [13] review the role of CSI showing how it has improved with advances in conformal techniques and is now being incorporated into preclinical models, suggesting that this approach may be used to address outstanding questions relating to the benefits of dose boosting following chronic treatment. Fernandez-Palomo et al. [14] review the current evidence for microbeam radiotherapy (MRT) in animal models. This approach uses spatially-modulated modulated microscale beam geometries delivered at high peak dose and dose rate, and has shown equivalent levels of tumour control compared to conventional beam deliveries, but with reduced toxicity in normal tissues.

Several papers also focus on recent advances in modelling tumour responses. For example, Cosper et al. [15] review the approaches, biological advantages and potential pitfalls of patient-derived models in head and neck cancer. In a corollary article, Vincent-Chong and Seshadri [16] present a novel syngeneic mouse model of oral squamous cell carcinoma derived from a chemically-induced tongue tumour in a C57BL/6 mouse. The RP-MOOC1 model has mutational and histological similarities to human disease and may prove to be a valuable platform for the evaluation of novel drug-radiotherapy combination strategies, including the use of immune-checkpoint inhibitors (ICIs). In addition, Nesseler et al. [17] highlight the interplay between tumour burden and the radiation immune response, and report on the impact of hypofractionation and anti-PD-1 therapy on directly irradiated and abscopal tumours in a bilateral syngeneic fibrosarcoma model. Hypofractionation resulted in the local regression of primary tumours and evoked an abscopal response only when PD-1 blockade was combined with radiotherapy.

The final paper in the issue is a departure from the clinical arena. McDonald and colleagues [18] describe the NASA GeneLab Platform that is being used for multi-omics analysis of biological responses to space radiation in animal models. This captivating study identified distinct biological signatures, associating specific ions with specific biological responses following space flight, and exemplifies how animal models can be used to better understand the human health risks from galactic cosmic rays.

In conclusion, all models are constrained by what is known, what is unknown, and what is assumed; animal models are no exception. Undoubtedly, such models will always fall short of capturing the complexities of the human patient population, so that significant care must be taken when choosing an appropriate and relevant model. Consideration of the requisite criteria should include not only pathological and physiological similarities, using both anatomy and parameters pertaining to the field of interest (causation factors, timeline of disease and progression, etc.), but also the known molecular and cellular response. To minimize potential confounders and limitations, researchers should choose a model based on as many relevant criteria as possible. For example, as described by Hopewell et al., when assessing a number of animal models to examine lung radiation effects, basing a choice purely on anatomical similarity to the human would lead to the use of the horse as a model [19]. Nonetheless, we believe that the articles presented in this edition underscore the potential benefits of using animal models; when used appropriately and with an understanding of their limitations, they can enable the translation of novel treatment paradigms to the clinic. We would like to thank all of the authors who have contributed their work and insightful opinions to this important issue.
